# Hybrid minimally invasive treatment of intralobar pulmonary sequestration: a single-centre experience

**DOI:** 10.1093/icvts/ivab245

**Published:** 2021-09-04

**Authors:** William Grossi, Francesco Londero, Alessandro Vit, Elisa De Franceschi, Gianluca Masullo, Massimo Sponza, Angelo Morelli

**Affiliations:** Department of Cardiothoracic Surgery, Thoracic Surgery Unit, Santa Maria della Misericordia Hospital, Udine, Italy; Department of Cardiothoracic Surgery, Thoracic Surgery Unit, Santa Maria della Misericordia Hospital, Udine, Italy; Department of Radiology, Interventional Radiology Unit, Santa Maria della Misericordia Hospital, Udine, Italy; Department of Cardiothoracic Surgery, Thoracic Surgery Unit, Santa Maria della Misericordia Hospital, Udine, Italy; Department of Cardiothoracic Surgery, Thoracic Surgery Unit, Santa Maria della Misericordia Hospital, Udine, Italy; Department of Radiology, Interventional Radiology Unit, Santa Maria della Misericordia Hospital, Udine, Italy; Department of Cardiothoracic Surgery, Thoracic Surgery Unit, Santa Maria della Misericordia Hospital, Udine, Italy

**Keywords:** VATS, Amplatzer, Embolization, Sequestration, Lobectomy

## Abstract

Pulmonary sequestrations are rare congenital malformations. They are often located in the lower lobes, and they are supplied by an aberrant systemic vessel arising from the thoracic aorta or abdominal arteries. These pulmonary malformations are divided into intra- and extralobar sequestrations, depending on the respective lack or presence of an independent pleural covering. Pulmonary sequestration can be asymptomatic or lead to recurrent pulmonary infections. The goal of this study was to analyse the feasibility and safety of a hybrid sequential approach. We report a small series of intralobar pulmonary sequestrations, from November 2017 to December 2018, successfully treated with a hybrid minimally invasive approach consisting of endovascular embolization of the aberrant arterial branch followed by video-assisted thoracoscopic lobectomy the day after. Thoracic pain following endovascular embolization was noted in all cases. Patients were discharged early in the absence of major postoperative complications. Prolonged air leak was observed in only 1 case. Despite the presence of sequestration-related pulmonary inflammation, in our experience, hybrid treatment for intralobar pulmonary sequestration is a safe and reproducible approach in terms of postoperative complications and hospital stay.

## INTRODUCTION

Pulmonary sequestrations (PS) are rare malformation of the lung, wherein a portion of the lung’s parenchyma is supplied by an aberrant vessel arising from the thoracic or abdominal aorta. Pulmonary sequestrations are divided into 2 main groups, intralobar PS (ILPS) and extralobar PS, depending on the presence of an independent visceral pleural covering. PS can be asymptomatic and revealed accidentally at radiological investigations or can lead to recurrent pulmonary infections in patients who present with cough, fever, chest pain and haemoptysis. ILPS accounts for about 75% cases of sequestrations [1]. Their vascular supply usually originates from the descending thoracic aorta and, less commonly, from the abdominal aorta, coeliac trunk or intercostal arteries [2]. Conversely, venous drainage is mostly guaranteed through the pulmonary venous system. Surgical resection is generally recommended in order to prevent ischaemic and infectious complications [1, 2]. However, despite the recent increase in the application of the thoracoscopic approach [video-assisted thoracoscopic surgery (VATS)] in lung surgery, there are still some concerns about the use of minimally invasive techniques for ILPS due to the risk of haemorrhage caused by incidental injury of the fragile aberrant artery wall and its retraction deep into the mediastinum or into the abdomen [3] during surgical manipulation. Although there have been several reports of the use of vascular plugs to exclude ILPS, mostly in paediatric patients [4], to the best of our knowledge there are only 2 reports in the literature about a hybrid sequential approach involving atypical lung resection [2], but none followed by major pulmonary resection.

## PATIENTS AND METHODS

We describe a small case series of 3 patients seen between November 2017 and December 2018 who underwent VATS lobectomy after endovascular embolization of the aberrant arterial vessel with an Amplatzer plug (Abbott Laboratories, Abbott Park, IL, USA). The goal of this study was to demonstrate that the hybrid approach previously described, endovascular and VATS, is feasible and safe, even in cases of pulmonary inflammatory disease and thick adhesions.

**Figure 1:**
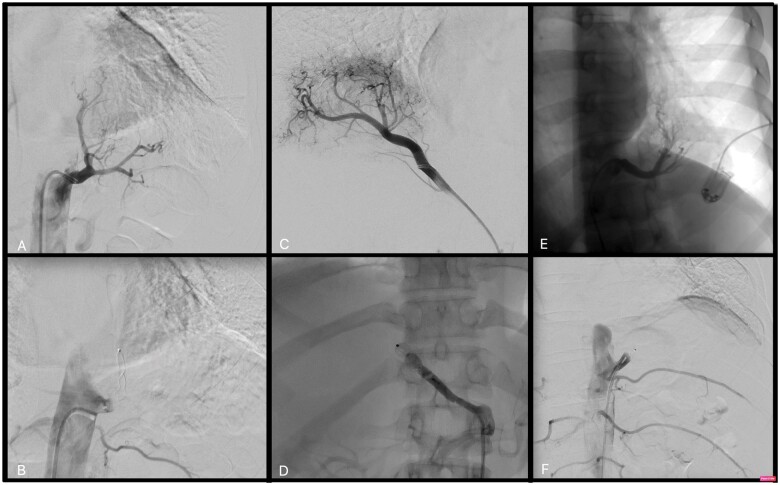
(**A**) Case 1: Transcatheter arterial angiography shows the aberrant branch supplying the left lower lobe originating from the thoracic aorta. (**B**) Case 1: The anomalous artery branch was catheterized with a guiding catheter, and it was then embolized by positioning an Amplatzer vascular plug type II, 12 mm. (**C**) Case 2: Transcatheter arterial angiography shows the aberrant branch supplying the right lower lobe originating from the coeliac trunk. (**D**) Case 2: The aberrant branch was successful embolized with an Amplatzer plug. (**E**) Case 3: Transcatheter arterial angiography shows the aberrant branch supplying the left lower lobe originating from the thoracic aorta. (**F**) Case 3: Endovascular embolization of the anomalous arterial branch of the descending thoracic aorta.

To perform the endovascular embolization procedure, the patients were placed in a supine position; the patient was given a local anaesthetic, the right femoral artery was punctured and a 5–7-Fr (depending on the anatomical situation) 11-cm-long sheath was inserted. Transcatheter arterial angiography revealed the aberrant branch originating from the thoracic aorta (2 cases, Fig. 1A–E) or from the abdominal aorta crossing the diaphragm (1 case, Fig. 1C). The anomalous artery branch was then catheterized with a guiding catheter, and the aberrant vessel was embolized by positioning a plug (Amplatzer vascular plug type II) 8, 10 and 12 mm in size, respectively (depending on the arterial size). Once the correct position of the plug was verified by angiography, it was deployed. A final angiographic check confirmed the occlusion of the vessel (Fig. 1B–F).

All VATS lobectomies were performed using the D’Amico technique [5]. Systemic arterial vessels were divided with a vascular stapler or by using high-energy devices after application of a Hem-o-lok clip. All patients had 1 chest tube (24 or 28 Ch) at −20 cmH_2_O suction for at least 24 h. The chest tube was removed when an air leak was ruled out and the fluid output was <200 cc. The patients were discharged the day after, depending on their degree of mobilization.

*Case 1*. A man, 58 years old, non-smoker, with a history of recurrent pulmonary infections. A chest computed tomography (CT) scan revealed 2 lesions located in the left lower lobe: a large basal ILPS (90 mm × 55 mm) supplied by a large aberrant vessel arising from the thoracic aorta and another lesion was represented by a typical carcinoid (36 mm × 30 mm). The patient underwent left lower lobe VATS lobectomy (operative time 205 min, blood loss, 50 ml). The postoperative period was uneventful. He was discharged on the fourth postoperative day.

*Case 2*. A woman, 43 years old, former smoker, with cough and sputum. Laboratory results revealed neutrophil leucocytosis and increased levels of C-reactive protein (CRP). A CT scan showed a large right lower lobe consolidation (85 mm × 55 mm) supplied by an aberrant arterial vessel arising from the coeliac trunk (Fig. 1C). High CRP levels and fever occurred after endovascular embolization (Fig. 1D), so preoperative intravenous therapy with piperacillin/tazobactam, 18 g per day, was administrated. After 6 days of antibiotic therapy, based on the decrease in the CRP levels, the patient had a right lower VATS lobectomy (operative time 215 min; blood loss, 100 ml). The postoperative period was uneventful, and the patient was discharged on postoperative day 6.

*Case 3*. A man, 31 years old, native of Bangladesh, was admitted to the pneumology unit with a high temperature and chest pain associated with left basal pulmonary consolidation on the chest x-ray. A CT scan revealed an area in the left lower lobe of the lung consistent with a large ILPS, evolving into a lung abscess, supplied by a large aberrant branch of the thoracic aorta. For this reason, the patient underwent intravenous antibiotic therapy preoperatively. The surgical treatment was temporarily halted until the CRP level returned to the suggested target. After 13 days, the patient had endovascular embolization (Fig. 1E and F) prior to a left lower VATS lobectomy (operating time 315 min, blood loss, 300 ml). The postoperative period was complicated by air leaks associated with subcutaneous emphysema. A bronchopleural fistula was excluded by bronchoscopy. The thoracic drainage was removed on postoperative day 9. He was discharged on postoperative day 10.

## DISCUSSION

The surgical management of PS by VATS is accepted worldwide and is superior to the thoracotomy approach [6, 7] in terms of postoperative outcome, hospital stay and postoperative pain. There are still some concerns about the management of haemorrhage following VATS in case of rupture of the aberrant vessel during dissection manoeuvres [3]. Therefore, we consider it reasonable to perform preoperative endovascular embolization of large aberrant vessel to obtain the aberrant systemic vessel’s occlusion, which may be friable due to chronic inflammation, with the aim to prevent potentially life-threatening intraoperative bleeding in course of surgery for major pulmonary resection [8]. Likewise, endovascular embolization is not a complication-free procedure. In fact, in the literature, the complications related to endovascular embolization, such as thrombosis, infections of the puncture site and migration of embolization material to non-target arteries have been widely reported, and they appear to be more frequent in the paediatric population. Meanwhile, in the adult population, the described sequelae mirror the side effects reported for similar endovascular procedures, such as bronchial embolization for haemoptysis or pulmonary arterio-venous malformation. The scariest complication related to endovascular embolization is inadvertent embolization of a spinal artery [9]. Placement of an Amplatzer plug was effective in terms of vessel occlusion and was safe with a low rate of device-related complications. Based on the work of other authors [2], we selected a plug that was ∼20–30% larger than the vessel diameter for secure fitting, to prevent dislocation or migration of the device. In all 3 cases treated in our thoracic unit, we observed thoracic pain after the endovascular embolization, a symptom that is common and widely described in the literature [10]. It usually appears within the first 48 h and responds well to analgesics. The incidence of this complication appears to be related to the size of the embolized vessel; it is present in up to 31% of patients in whom the occluded pulmonary arterio-venous malformation has feeding vessels larger than 8 mm in diameter. In contrast with other authors who performed surgery 4 weeks after the embolization procedure [2], we performed VATS lobectomy the day after the endovascular embolization. In all 3 cases reported, the surgical indication was based on the ILPS size, ILPS superinfection or, in 1 case, association with lung cancer. In our experience, the surgical procedure was postponed in cases of ILPS superinfection, and the patients underwent surgery after they had shown full recovery from clinical symptoms and laboratory tests.

## CONCLUSION

Based on our initial experience, hybrid treatment of ILPS by VATS lobectomy after endovascular embolization was a safe and reproducible procedure, especially when performed by a skilled VATS surgeon, even in case of ILPS superinfection. Adhesions and lymphadenomegaly did not lead to a conversion. All patients reported intense thoracic pain after endovascular embolization, probably due to acute ischaemia of the aberrant vessel and its calibre; this symptom was not considered a complication related to endovascular embolization because of its fast remission after administration of painkillers. To our knowledge, we report the first case series of patients, even if small (3 cases treated), undergoing a safe hybrid sequential approach for symptomatic ILPS, represented by endovascular embolization with an Amplatzer plug prior to VATS lobectomy. However, ILPS is a rare entity, which so far has only been the subject of case reports in the literature.

## ETHICAL STATEMENT

The authors are accountable for all aspects of the work in ensuring that questions related to the accuracy or integrity of any part of the work are appropriately investigated and resolved.

**Conflict of interest:** none declared.

## Reviewer information

Interactive CardioVascular and Thoracic Surgery thanks Tevfik Kaplan and Vanessa Diaz-Ravetllat for their contribution to the peer review process of this article.
